# Flexible Bronchoscopy Combined With an Airway Maneuver for Foreign Material Removal Under Invasive Mechanical Ventilation

**DOI:** 10.7759/cureus.44239

**Published:** 2023-08-28

**Authors:** Georgios E Zakynthinos, Athanasios Mpaggeas, Konstantina Deskata, Christos Doudakmanis, Demosthenes Makris

**Affiliations:** 1 Third Department of Cardiology, Athens Chest Disease Hospital "Sotiria", Athens, GRC; 2 Department of Neurology, University Hospital of Larissa, Larissa, GRC; 3 Department of Critical Care, University General Hospital of Larissa, Larissa, GRC; 4 Department of Surgery, University Hospital of Larissa, Larissa, GRC; 5 Department of Critical Care, General University Hospital of Larissa, Larissa, GRC

**Keywords:** aspiration, airway obstruction, emergency care, invasive mechanical ventilation, flexible bronchoscopy

## Abstract

We present a case of a patient who had aspirated a massive amount of food, leading to cardiac arrest, and had to be intubated because of severe hypoxemia. The hypoxemia persisted, regardless of the recruitment maneuvers, performance of flexible bronchoscopy, and suctioning through the ventilating tube, because we were unable to reach the left main bronchus (LMB), where the greatest amount was concentrated. However, we managed to overcome this problem by using a prototype handling technique to catheterize the LMB directly with the usage of the flexible bronchoscope. We introduce this handling technique for the removal of foreign material from the LMB, which will probably be really useful in emergency situations, because of its simplicity and effectiveness.

## Introduction

Overall, foreign body (FB) aspiration is a condition commonly encountered in children and rarely in adults. Nevertheless, it can indeed occur in adults, often presenting as either respiratory failure or respiratory symptoms such as wheezing, coughing, and breathlessness. Foreign bodies can encompass a wide array of objects, broadly categorized as organic (e.g., food or bones) and inorganic (e.g., teeth and plastic objects) [1].

Diagnosing FB aspiration can be particularly challenging, necessitating a high degree of clinical suspicion and radiological examinations. Bronchoscopy plays a pivotal role in both diagnosis and treatment, enabling the direct visualization and potential removal of the foreign body. While rigid bronchoscopy is commonly the preferred approach for children, flexible bronchoscopy has gained prominence in adult cases, accounting for approximately 80% of procedures in certain reports [2]. The benefits of flexible bronchoscopy lie in its ease, safety, and brevity, particularly when performed by experienced practitioners. These attributes render it invaluable, especially in emergency scenarios involving respiratory failure caused by foreign body obstruction.

However, what unfolds when the foreign body in question is a substantial quantity of food lodged within the left main bronchus (LMB), seemingly inaccessible for conventional removal? Herein, we present a case of food material aspiration, further complicated by cardiac arrest, and its successful management through a combination of bronchoscopy and an innovative airway maneuver.

## Case presentation

A 55-year-old male was admitted to the intensive care unit (ICU) following the successful resuscitation of a cardiac arrest resulting from the aspiration of a substantial amount of food. His medical history included schizophrenia and epilepsy. Upon admission, he was intubated and connected to mechanical ventilation with the following settings: tidal volume of 450 mL, positive end-expiratory pressure (PEEP) of 6 cmH_2_O, fraction of inspired oxygen of 100%, and plateau pressure of 24 cmH_2_O. His body temperature was recorded as 35°C, with a respiratory rate of 25 breaths/minute, pulse rate of 110 beats/minute, and blood pressure of 90/40 mmHg.

**Figure 1:**
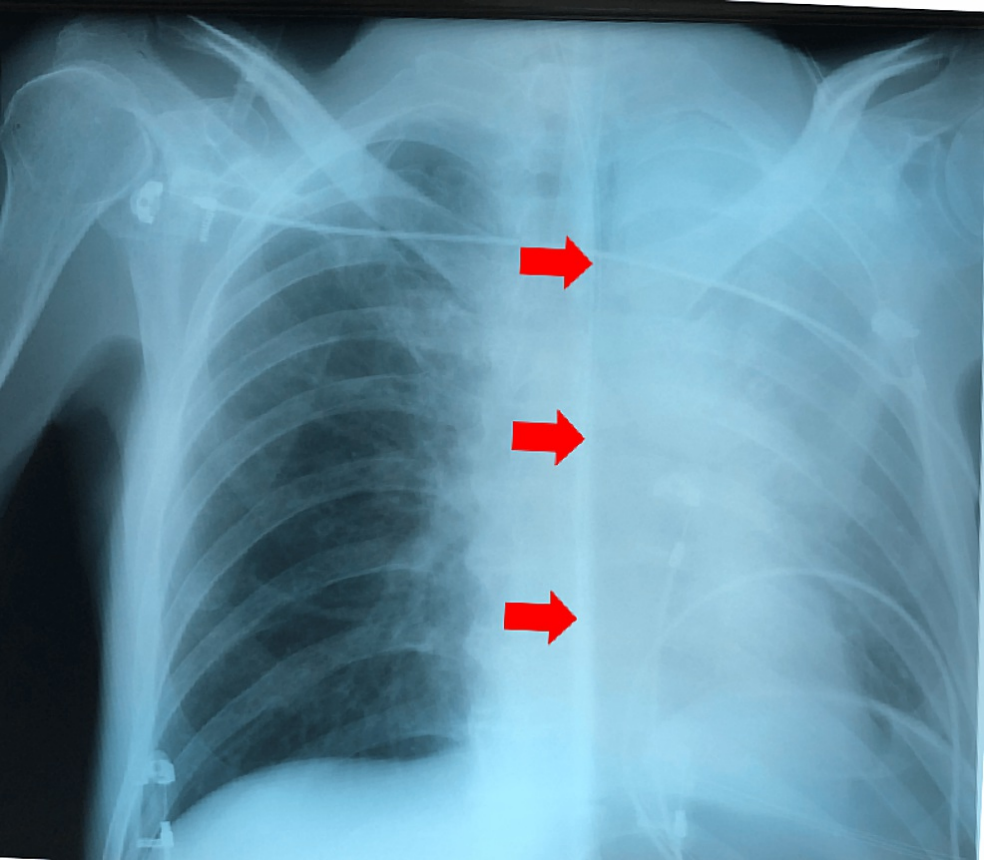
Initial chest X-ray upon admission A chest X-ray showed a nearly total opacification of the left hemithorax, implying a full atelectasis of the left upper lobe and a partial atelectasis of the left lower lobe, loss of cardiac silhouette, and displacement of the mediastinum to the left (red arrows)

The patient exhibited profound arterial hypoxemia coupled with hypercapnic acidosis (arterial oxygen saturation {SpO_2_} of 54%, arterial blood pH of 7.19, bicarbonate {HCO_3_} of 23.7 mmol/L, partial pressure of oxygen {pO_2_} of 57 mmHg, and partial pressure of carbon dioxide {pCO_2_} of 62 mmHg). Clinical examination findings revealed diminished left lung sounds and restricted expansion of the left thoracic wall. A chest X-ray demonstrated the opacification of the left hemithorax (Figure 1).

**Figure 2:**
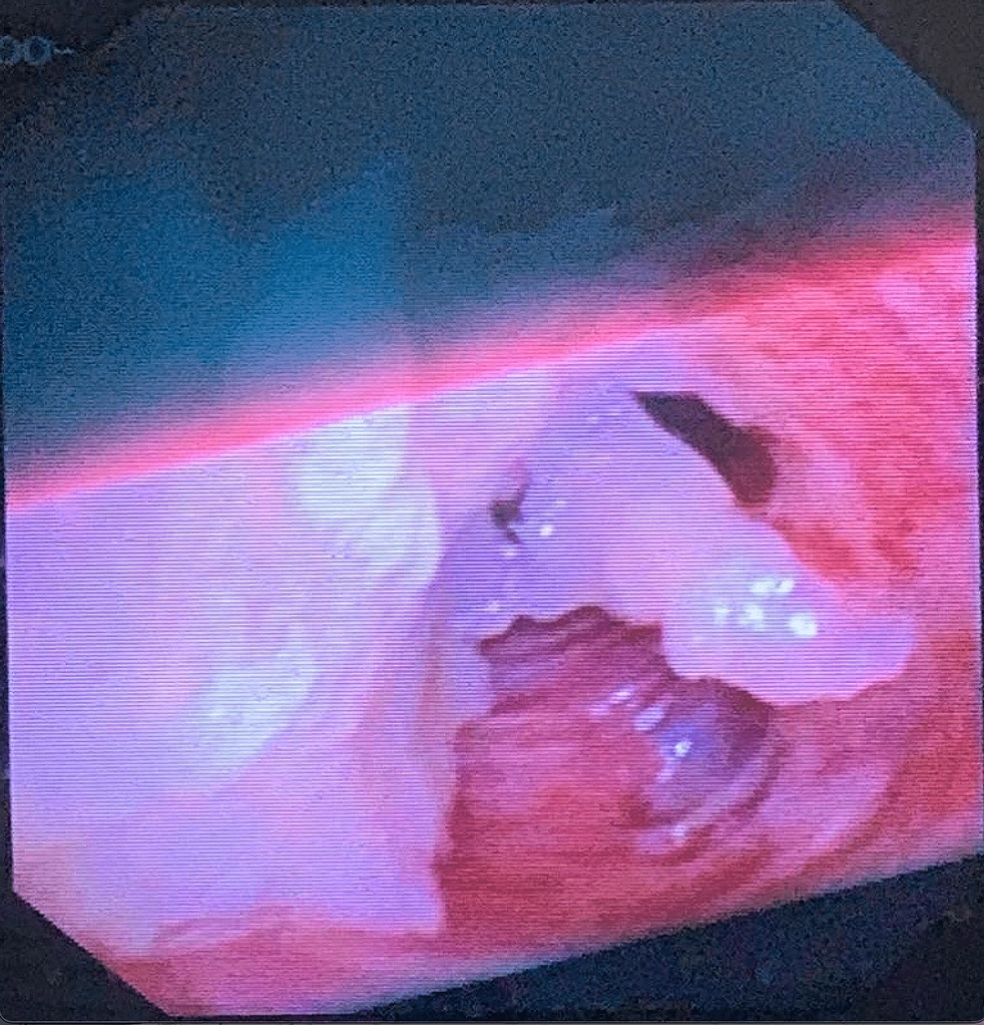
LMB obstructed with food LMB: left main bronchus

Despite employing recruitment maneuvers, elevating PEEP, and conducting bronchial suction through the ventilating tube, persistent hypoxemia remained (SpO_2_ of 64%). In response, the patient was promptly repositioned laterally, with the left side elevated. Following this, a flexible video bronchoscopy using an Olympus Exera-III-BF1T-180 bronchoscope (Tokyo, Japan) was performed. This procedure unveiled a significant quantity of recently aspirated food predominantly located within the main airways, notably concentrated in the left main bronchus (LMB) (Figure 2).

Despite concerted efforts to aspirate the foreign body (FB) using both the bronchoscope (3.0 mm working channel) and a suction tube (4 mm) through the ventilating circuit, a substantial portion of the FB persisted within the LMB. The anatomical positioning rendered it challenging to access and extract the FB using conventional methods.

**Figure 3:**
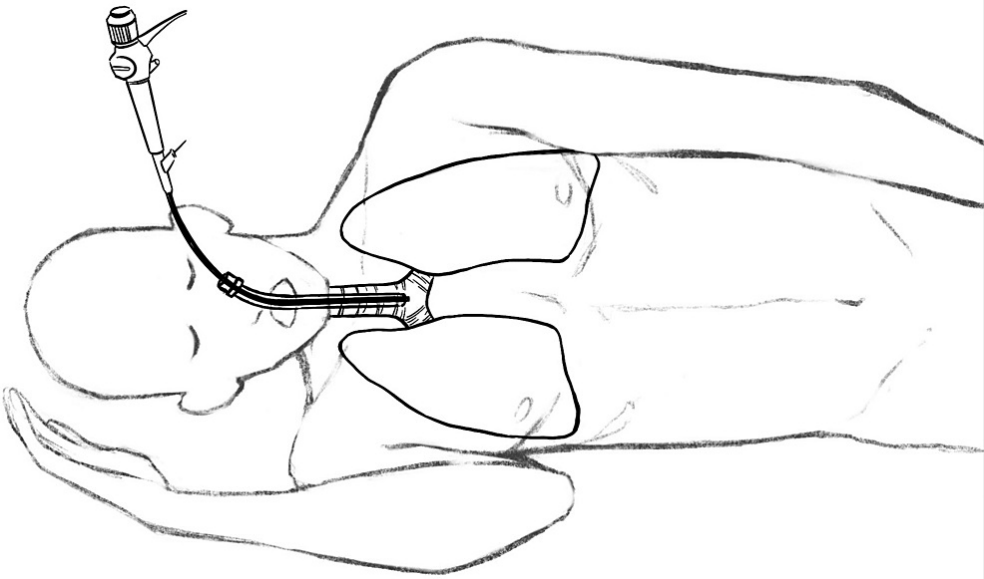
The patient in lateral position before the handling (illustrated by one of the authors)

**Figure 4:**
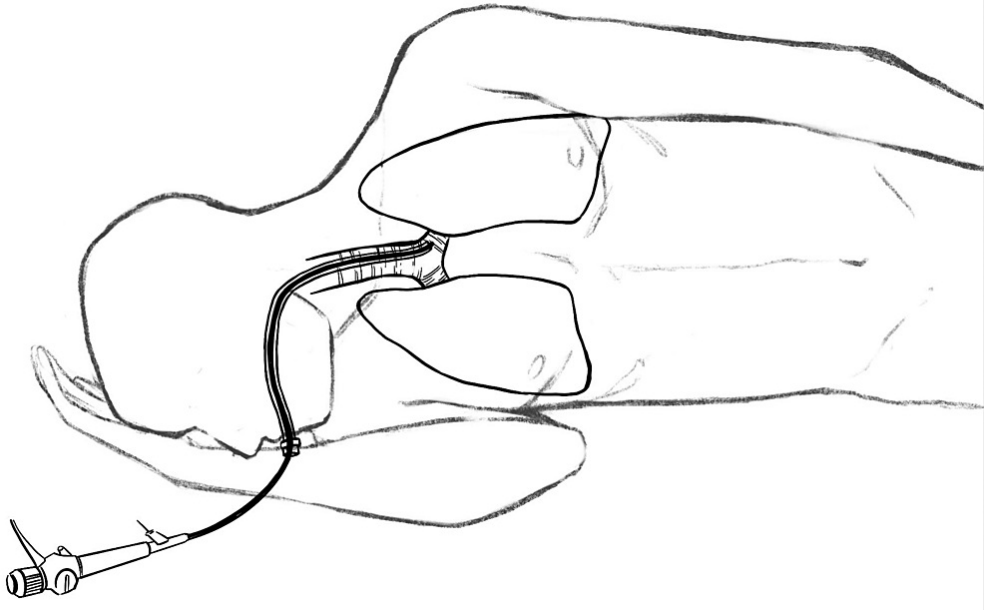
The described handling (illustrated by one of the authors)

To address this challenge, the LMB was catheterized using the ventilating tube. This involved passing the bronchoscope through the ventilating tube, advancing it into the LMB, and subsequently advancing the ventilating tube (with the cuff deflated) within the LMB. Importantly, achieving successful catheterization of the LMB by the ventilating tube necessitated the further rotation of the patient's head toward the right side (Figures 3, 4). This strategic positioning was essential to prevent the ventilating tube from causing undue pressure on the flexible bronchoscope, thus facilitating advancement toward the LMB.

**Figure 5:**
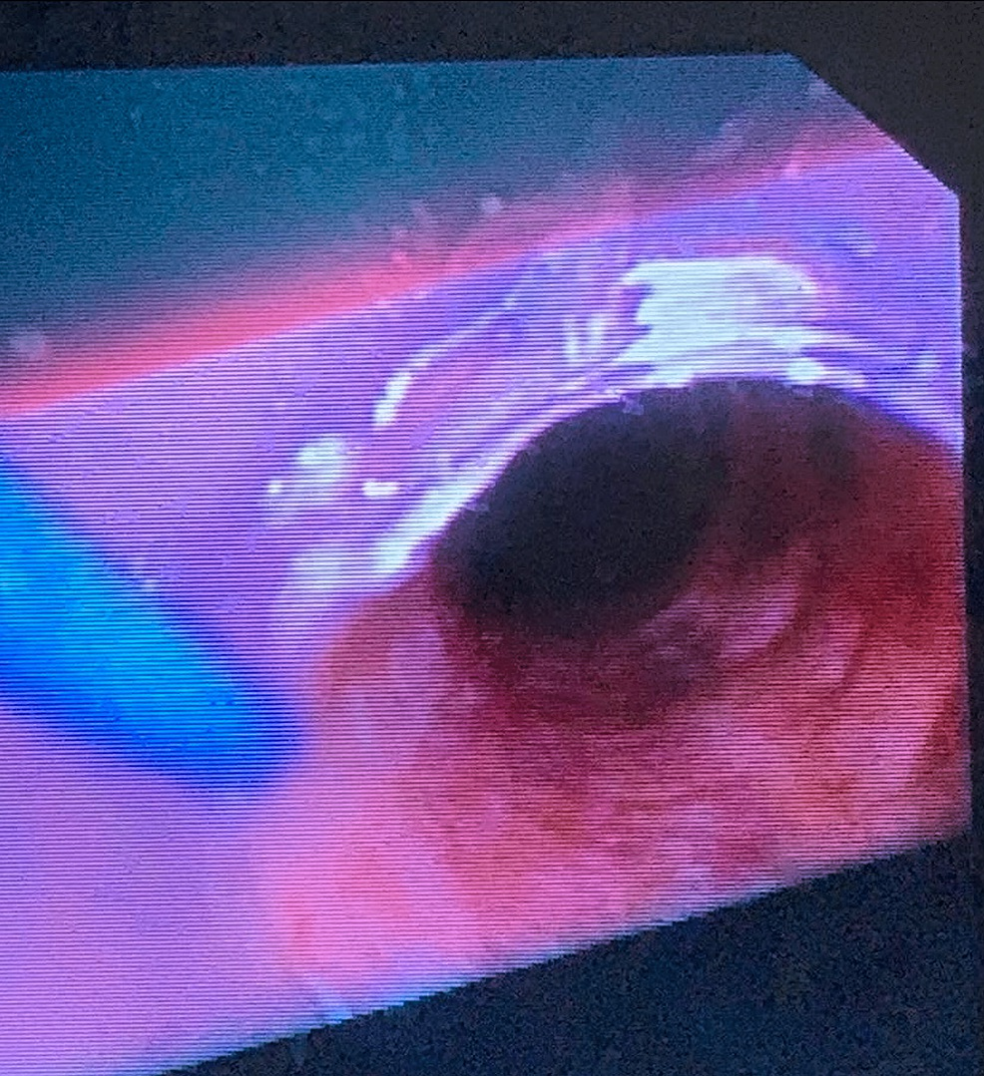
The trachea after aspiration using the prescribed handling

This intervention led to the proper positioning of the suction tube within the left main bronchus (LMB), enabling the successful removal of the foreign body (FB) (Figure 5). The entire procedure was completed in approximately three minutes, resulting in the restoration of normal arterial oxygen saturation. Notably, during this brief timeframe, a significant episode of hypoxemia emerged, aggravated by the predominant distribution of the tidal volume to the left lung. This juncture proved to be critical, prompting a temporary suspension of the procedure. During this pause, rapid and essential adjustments were executed, involving the repositioning of the intubation tube to its accurate placement. With these adjustments seamlessly implemented, the procedure resumed its course. It is essential to underscore that the instances of hypoxemia require careful consideration due to their potential occurrence during the procedure, highlighting the importance of vigilant monitoring and timely intervention to ensure patient safety. Following the intervention, the patient was administered antibiotics and underwent a 24-hour cooling protocol, as recommended post cardiac arrest. Remarkably, the patient exhibited a remarkable recovery and was discharged just three days later, achieving a Glasgow Coma Scale score of 15.

## Discussion

The present case illustrates how combining endoscopy with a straightforward airway maneuver in an emergency setting can facilitate the removal of a foreign body (FB) obstructing the main airway, thus preventing asphyxia in intubated patients.

In our case, the patient aspirated a substantial amount of food, leading to the obstruction of the main airways, specifically the left main bronchus (LMB). In such scenarios, the working channel of a flexible endoscope might be too narrow to effectively aspirate the FB, and a standard suction tube may not reach the site of obstruction. To overcome this challenge, we utilized a prototype handling technique. Initially, we used the bronchoscope as a guide to position the suction tube through the ventilating tube into the LMB. This was facilitated by positioning the patient laterally (left side upward) and turning their head to the right, allowing the easy insertion of the bronchoscope to the left and subsequently threading the ventilating tube into the LMB.

Without the utilization of this technique and with the patient remaining supine, the ventilating tube could overpower and bend the bronchoscope, causing it to move toward the right main bronchus. This handling approach necessitates the coordinated efforts of two operators: one managing the bronchoscope and the other handling the patient's head and the ventilating tube. Using this technique, we successfully removed the FB and aided the patient's recovery.

An alternative approach could have been the use of a rigid bronchoscope, offering advantages such as a larger working channel, the availability of sizable forceps, and the simultaneous use of multiple instruments. However, in emergency situations involving unknown causes of respiratory failure, especially when mechanical ventilation is essential, a fast-track approach such as immediate flexible bronchoscopy is crucial to prevent severe complications associated with asphyxia [1]. Previous studies indicate that flexible bronchoscopy is the preferred initial option, reporting success rates ranging from 60% to 100% [2-5]. Additionally, flexible bronchoscopy boasts a higher success rate, lower complication rate, and shorter procedure duration when compared to rigid bronchoscopy [6]. These factors make flexible bronchoscopy more suitable for emergent cases, where time is of the essence, and procedural mistakes are more likely to occur. Complication rates for rigid bronchoscopy vary between 3.4% and 13.4%, contingent on the expertise of the invasive pulmonologist, with this risk escalating during emergent procedures [7]. In contrast, Wiemers et al. noted a similar overall complication rate between rigid and flexible bronchoscopy (19.1% versus 24.2%, p = 0.232), with higher respiratory complications associated with flexible bronchoscopy (9.2% versus 16.3%, p = 0.025). However, in cases with comprehensive respiratory monitoring and a secure airway, as seen in our intubated patient, flexible bronchoscopy proves to be a safe procedure [8].

An alternative solution might have involved placing the patient in a position that facilitates FB removal, such as reverse Trendelenburg, as reported previously [9]. However, this approach may be more suitable for less severe cases of intubated patients not exhibiting signs of asphyxia. Fang et al. described a case involving an 11-year-old patient who had aspirated a FB larger than the diameter of an available rigid bronchoscope. In their case, a flexible bronchoscope was used in conjunction with a laryngoscope for FB removal [10]. In our case, the role of the laryngoscope was substituted by the intubation tube, which served as a guide. Furthermore, the presence of the intubation tube rendered the use of a laryngoscope unnecessary, as the problem in our case was identified as the bronchoscope's inability to reach the LMB due to anatomical constraints, rather than limitations in the bronchial tree's diameter.

Another often-suggested approach involves the use of medications to prevent laryngospasm, such as intranasal dexmedetomidine [11]. However, in our case, the patient was already heavily sedated, negating the risk of laryngospasm. Moreover, the issue we faced was not due to the bronchial tree's limited diameter but rather the bronchoscope's inability to access the LMB due to anatomical reasons.

## Conclusions

In conclusion, we propose that the insertion of the suction tube through the ventilating tube into a main bronchus, facilitated by flexible bronchoscopy and an airway maneuver, represents a feasible option for the removal of foreign objects obstructing the left main bronchus (LMB). This technique holds the potential to prevent life-threatening hypoxic events during emergencies.

However, it is important to emphasize that this procedure should not be considered for routine application and demands a high degree of caution. It is a specialized intervention that requires the expertise of an experienced invasive pneumonologist. This approach becomes particularly valuable when conventional methods have proven ineffective or are unavailable, offering a potentially life-saving alternative in critical situations.
